# Factors Influencing Self-Care Behavior and Treatment Adherence in Hemodialysis Patients

**DOI:** 10.3390/ijerph182412934

**Published:** 2021-12-08

**Authors:** Hana Kim, Mi-Kyoung Cho

**Affiliations:** Department of Nursing Science, School of Medicine, Chungbuk National University, Cheongju 28644, Korea; highting1@naver.com

**Keywords:** hemodialysis, kidney failure, self-care, social support, treatment adherence

## Abstract

Low self-care and treatment adherence are found among hemodialysis patients. We aimed to identify the factors influencing self-care behavior and treatment adherence and examine the mediating effect of treatment adherence on self-care behavior. A questionnaire was administered through a social media community from 11 July to 13 August 2021. The data collected from 100 participants were analyzed using the independent *t*-test, one-way analysis of variance, Pearson’s correlation, multiple linear regression analysis, and hierarchical multiple regression analysis. The mean self-care behavior and treatment adherence scores were 3.52 ± 0.57 and 4.01 ± 0.48, respectively. The mean age and hemodialysis duration were 51.70 ± 9.40 and 7.57 ± 7.21 years, respectively. The common primary cause of end-stage renal disease was glomerulonephritis (*n* = 39, 39%). Self-care behavior varied with education, frequency of self-care behavior education, and social support and was positively correlated with treatment adherence and social support. Treatment adherence was positively correlated with social support. Treatment adherence, social support, and health status were influenced self-care behavior (54.5%. Self-care behavior and frequency of self-care behavior education influenced treatment adherence (61.3%). Treatment adherence partially mediated the relationship between social support and self-care behavior. Intervention strategies that increase both social support and treatment adherence can promote self-care behavior.

## 1. Introduction

The incidence of chronic renal failure is increasing aging population and the rising prevalence of chronic diseases. These patients undergo renal replacement therapy (RRT), such as hemodialysis (HD), peritoneal dialysis, and kidney transplantation, due to the loss of kidney function. The number of patients undergoing RRT in South Korea is continuously on the rise, increasing from 28,046 in 2000 to 108,873 in 2019; HD accounted for 83.6% of all RRTs, showing that HD is the most common RRT [1]. Once initiated, HD must be maintained for the rest of the patient’s life unless the patient receives a kidney transplant.

Self-care among HD patients refers to activities that promote survival, functional integration, and well-being [2]. These include diet management, arteriovenous fistula (AVF) management, medication administration, exercise, blood pressure and weight control, and physical management. HD patients must continue engaging in self-care to successfully manage their condition, prevent and manage acute and chronic complications, and enhance their quality of life [3]. Self-care is often challenging, as it requires patients to strictly control themselves for the remaining years of their lives [4].

Previous studies on self-care practice showed that patients easily display self-care behaviors pertaining to medication and fistula management [4,5,6]. However, there is poor display of certain behaviors, such as management of blood pressure and body weight [4,7,8] and social adjustment [4,5,7]. These studies showed better implementation of self-care behavior when directly related to the treatment. However, the patients tend to poorly implement self-care behavior with relatively lower impact on the treatment.

Treatment adherence refers to the act of following the medical prescriptions given by a health care provider [9]. Treatment adherence among HD patients is critical to their health management [10,11]. It encompasses adhering to the HD treatment, fluid and diet restrictions, and medication [12]. Many studies reported that the highest patient adherence was to the HD treatment, followed by medication [4,12,13,14,15]. Treatment nonadherence is a rampant problem among HD patients [14,16,17]. In particular, the rate of nonadherence to diet and fluid restrictions, which has little direct impact on the treatment, ranges from 40% to 80% [12,17].

In a narrow sense, treatment adherence is considered passive self-care behavior. Self-care is a broad concept that encompasses treatment adherence and connotes more active self-care behavior. Continuously engaging in active self-care behavior—with passive self- care behavior as the basis—is critical in chronic diseases. The relationship between self-care behavior and HD nonadherence, as a type of treatment nonadherence [7], has been investigated; however, most of these studies examined either treatment adherence and self-care behavior separately. Studies simultaneously examining self-care behavior and overall treatment adherence are lacking. Hence, there is a need to identify the factors influencing self-care behavior among HD patients by concurrently examining treatment adherence and self-care behavior and to develop interventions accordingly. This would contribute to formulating nursing strategies to promote self-care behavior among HD patients.

The aim of this study was to identify the factors influencing self-care behavior and treatment adherence in HD patients. In addition, the study aimed to confirm the mediating effect of treatment adherence in the relationship between social support and self-care behavior, which could be used as a reference for the construction of a self-care promotion program for hemodialysis patients in the future. The specific purposes included, first, to identify the participant’s characteristics, self-care behavior, treatment adherence, and social support of hemodialysis patients; second, to investigate the correlation between self-care behavior, treatment adherence, and social support of hemodialysis patients; third, to identify the factors influencing self-care behavior and treatment adherence of hemodialysis patients; and finally, to confirm the mediating effect of treatment adherence between the social support of hemodialysis patients and their self-care behavior.

## 2. Materials and Methods

### 2.1. Study Design

This was a descriptive survey aiming to identify the factors influencing self-care behavior and treatment adherence among HD patients.

### 2.2. Study Population and Sampling

The study population comprised members of social media communities (Kidney patients’ community, Nationwide kidney patients’ community in Naver Band, and Community for patients with kidney diseases in Daum Cafe) who periodically undergo HD. The specific inclusion criteria were:

1. Patients aged ≥18 years who were diagnosed with end-stage renal disease (ESRD) and had undergone HD for at least one month;
2. HD for at least twice a week;
3. Ability to comprehend the Korean language and complete an online questionnaire; and
4. Provision of informed consent to participate in the study.

Participants who withdrew their consent during the study and those hospitalized for other complications were excluded. The sample size was determined using the G*power 3.1 software based on a previous study [5]. For a regression analysis with an effect size of 0.33, significance level of 0.05, power of 0.80, and 11 affected variables (age, sex, marital status, education, occupation, economic status, health status, primary cause of ESRD, duration of HD, frequency of self-care behavior education, and social support), the minimum sample size was calculated to be 87. In consideration of a 10% withdrawal, 97 participants were recruited; however, 100 participants completed the online questionnaire (link provided on the community board) and were included in the analysis.

### 2.3. Instruments

#### 2.3.1. Participant Characteristics

Ten characteristics were analyzed, including age, sex, marital status, education, occupation, and economic status. The remaining four characteristics were disease-related, namely health status, primary cause of ESRD, duration of HD, and frequency of self-care behavior education.

#### 2.3.2. Self-Care Behavior

Self-care among HD patients refers to activities that promote survival, functional integration, and well-being [2]. Self-care behavior was measured using the tool for HD patients developed by Song [3] and modified and adapted by Cho and Choe [4]. This 35-item tool comprises six items for diet, six items for fistula management, four items for exercise and rest, two items for medication, two items for management of blood pressure and body weight, four items for social adjustment, and eleven items for management of physical problems. Each item is rated on a five-point Likert scale, from 1 (“I don’t follow the recommendations at all”) to 5 (“I always follow the recommendations”). The total score ranges from 35–175, and a higher score indicates better self-care behavior. The reliability (Cronbach’s α) of the tool was 0.86 in the study by Cho and Choe and 0.931 in this study [4].

#### 2.3.3. Treatment Adherence

Treatment adherence refers to the act of following medical prescriptions by health care providers [9]. Treatment adherence was measured using a Korean translation of the Hemodialysis Treatment Adherence Questionnaire developed by Indino et al. [12]. The content validity of the scale was established by the developer, and that of the translated scale was assessed by six relevant experts (nurses with more than 10 years of experience in an artificial kidney unit). The mean content validity index was 1.0. This 11-item tool comprises two items for HD treatment, three items for medication, three items for fluid restrictions, and three items for diet. Each item is rated on a five-point Likert scale from 1 (“almost always”) to 5 (“never”). The total score ranges from 11 to 55, and a higher score indicates greater treatment adherence. The Cronbach’s α was 0.636 in this study.

#### 2.3.4. Social Support

Social support is defined as the resources that help an individual feel loved, cared for, respected, valued and as part of an organization through which they can communicate and have mutual responsibility [18]. It serves as a buffer for stress in various disease-related situations and thus alleviates adverse health outcomes [19]. The Multidimensional Scale of Perceived Social Support was developed by Zimet et al. and adapted by Shin and Lee [20,21]. This 12-item scale assesses social support based on support from family, friends, and significant others. Since our study was conducted among patients, we designated “significant others” as health care providers. Each item is rated on a five-point Likert scale from 1 (“strongly disagree”) to 5 (“strongly agree”). The total score ranges from 12 to 60, and a higher score indicates greater social support. The reliability (Cronbach’s α) of the scale was 0.89 in the study by Shin and Lee and 0.935 in this study [21].

### 2.4. Data Collection

This study was approved by the Institutional Review Board of Chungbuk National University in July 2021 (No: CBNU-202107-HR-0089). Prior to recruiting study participants, we asked for cooperation from the administrators of the targeted social media communities (Kidney patients’ community, Nationwide kidney patients’ community in Naver Band, and Community for patients with kidney diseases in Daum Cafe). We then posted a recruitment announcement to recruit participants from 11 July to 13 August 2021. The poster in the websites announcing recruitment of patients explained the study, including the purpose and method, guarantee of anonymity, strict use of the collected data only for research purposes, and freedom to refuse or stop participation at any time. If research participants wanted to withdraw their consent, they were asked to notify the researcher of their intention to withdraw through e-mail or phone call. Those who were willing to participate in the study were instructed to access the online questionnaire via Google Forms by clicking the provided link. The online questionnaire also explained the study and requested for consent before a participant was allowed to proceed with the questionnaire (81 items, 15 min). All participants were given a gift by draw.

### 2.5. Data Processing and Analysis

The collected data were analyzed using IBM SPSS, version 26.0 (IBM, Armonk, NY, USA). Participants’ general characteristics and social support were analyzed as the frequency and percentage or mean and standard deviation, and self-care behavior and treatment adherence were analyzed using descriptive statistics (mean and standard deviation). Normality test was performed using Kolmogorov–Smirnov test. Differences in the study parameters according to participant characteristics were analyzed using the independent *t*-test and one-way analysis of variance followed by the Scheffe test for post-hoc comparison. Correlations among the study parameters were analyzed using Pearson correlational analysis, and the variables confirmed to significantly differ in relation to self-care behavior and treatment adherence were entered into multiple linear regression analyses. The mediating effect of social support and treatment adherence on self-care behavior was analyzed using hierarchical regression, and the significance of the mediation was confirmed using the Sobel test [22]. Statistical significance was set at *p* < 0.05.

## 3. Results

### 3.1. Participant Characteristics

The mean age was 51.70 ± 9.40 years, and most of the participants were aged ≤49 years. Seventy-seven participants (77%) were married, 51 (51%) had a bachelor’s degree or higher, and 55 (55%) were unemployed. Forty-seven participants (47%) had low economic status. The most common primary cause of ESRD for HD was glomerulonephritis (*n* = 39, 39%). The mean health status score was 2.92 ± 0.96, with most of the participants (*n* = 47, 47%) rating their health as “moderate.” The mean HD duration was 7.57 ± 7.21 (Q1 = 2.27, Q2 = 5.08, Q3 = 9.95) years, and the annual average frequency of self-care behavior education was 8.17 ± 16.27. Twenty-three participants (23%) stated that they had one educational session per year. Most (*n* = 70, 70%) of the participants had moderate social support (Table 1).

### 3.2. Self-Care Behavior and Treatment Adherence

The mean self-care behavior score was 3.52 ± 0.57. The highest score was for medication, followed by fistula management. The scores for social adjustment and management of blood pressure and body weight were low. The mean treatment adherence score was 4.01 ± 0.48, with the highest and lowest scores for HD treatment and fluid restrictions, respectively (Table 2).

### 3.3. Differences in Self-Care Behavior and Treatment Adherence according to Participant Characteristics

Prior to the analysis, normality test was performed using Kolmogorov–Smirnov test of continuous variables (age, health status, duration of hemodialysis, frequency of self-care behavior education, social support, treatment adherence, and self-care behavior). The assumption of normality was fulfilled. Self-care behavior significantly differed according to education (F = 5.06, *p* = 0.008), health status (F = 9.20, *p* < 0.001), and duration of HD (F = 2.82, *p* = 0.043). Self-care behavior was higher among participants with college or higher education than among those with middle school or lower education, higher among those with “good” health status than among those with “moderate” or “poor” health status, and higher among those who had been on HD for 1–2.99 years than among those who had been on HD for < 1 year (Table 3). Treatment adherence significantly differed according to education (F = 9.97, *p* < 0.001), frequency of self-care behavior education (F = 4.22, *p* = 0.020), and social support (F = 3.905, *p* = 0.023). Treatment adherence was higher among participants with college or higher education than those with middle school or lower and high school education, higher among those who had at least two self-care behavior education sessions per year than among those without regular self-care behavior education, and higher among those with high social support than among those with low social support (Table 3).

### 3.4. Correlations among Self-Care Behavior, Treatment Adherence, Social Support

Treatment adherence (r = 0.62, *p* < 0.001) and social support (r = 0.56, *p* < 0.001) increased with increasing self-care behavior. Furthermore, social support (r = 0.33, *p* = 0.001) increased with increasing treatment adherence.

### 3.5. Influencing Factor of Self-Care Behavior and Treatment Adherence

To identify the influencing factor of self-care behavior and treatment adherence, participant characteristics that were significant in relation to self-care behavior and treatment adherence were analyzed using multiple linear regression. Treatment adherence, social support, education, health status, and duration of HD were entered in the regression equation for self-care behavior. Self-care behavior, social support, education, and frequency of self-care behavior education were entered in the regression equation for treatment adherence. Nominal variables were dummy-coded, and continuous variables were entered as is. Residual analysis using a histogram and normal P-P plot confirmed that the assumptions of normality and homogeneity of variance were fulfilled. Multicollinearity was tested based on tolerance and the variance inflation factor (VIF). Multicollinearity was confirmed to be absent, with a tolerance > 0.1 (0.21–0.99; 0.19–0.97) and VIF < 10 (1.01–4.88; 1.03–5.30). The independence of residuals was tested using the Durbin–Watson test and was confirmed with a Durbin–Watson statistic close to 2 (1.88 and 2.04).

The regression model for self-care behavior was statistically significant (F = 20.74, *p* < 0.001). Treatment adherence (**t** = 5.94, *p* < 0.001), social support (**t** = 4.16, *p* = 0.007), and health status (**t** = −2.45, *p* = 0.016) were significant influencing factor of self-care behavior. However, education and duration of HD were not statistically significant.

The regression model for treatment adherence was statistically significant (F = 17.47, *p* < 0.001). Self-care behavior (**t** = 4.34, *p* < 0.001) and frequency of self-care behavior education (**t** = 3.47, *p* = 0.001) were significant influencing factors of treatment adherence. However, social support and education were not statistically significant (Table 4).

### 3.6. Mediating Effect of Treatment Adherence on the Relationship between Social Support and Self-Care Behavior

**Figure 1:**
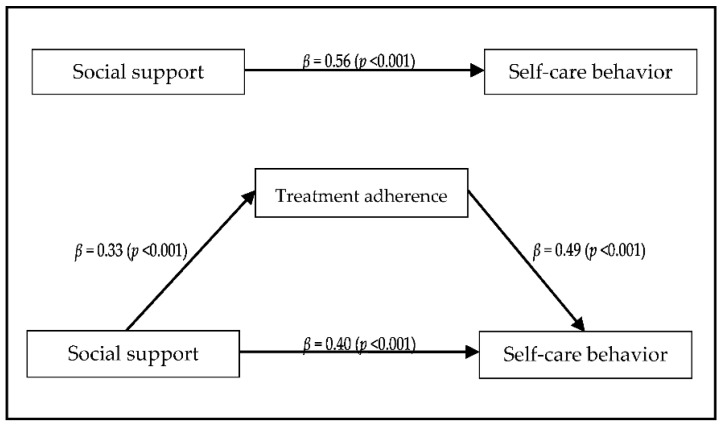
Mediating effect of treatment adherence on the relationship between social support and self-care behavior.

We performed three-step hierarchical regression analysis as outlined by Baron and Kenny to examine the mediating effects of treatment adherence on the relationship between social support and self-care behavior [23]. The Sobel test (Sobel) was used to confirm whether treatment adherence was a significant mediator [22]. In Step 1, the regression model testing the effect of the independent variable (social support) on the mediator (treatment adherence) was statistically significant. Treatment adherence increased with increasing social support (β = 0.33, *p* = 0.001). In Step 2, the independent variable (social support) had a statistically significant effect on the dependent variable (self-care practice). Self-care behavior increased with increasing social support (β = 0.56, *p* < 0.001). In Step 3, the mediator (treatment adherence) had a statistically significant effect on the dependent variable (self-care behavior) after adjusting for the independent variable (social support) (β = 0.49, *p* < 0.001). The β value (0.40) of the independent variable (social support) was smaller than the β value (0.56) of Step 2. This confirmed that treatment adherence had a partial mediating effect (Figure 1). The Sobel test [22] confirmed that the mediating effect of treatment adherence on the relationship between social support and self-care behavior was statistically significant (Z = 3.27, *p* = 0.001; Figure 1). Specifically, the treatment adherence of HD patients increased with increasing social support, and self-care behavior increased with increasing treatment adherence.

## 4. Discussion

This study was conducted to identify factors influencing self-care behavior and treatment adherence in hemodialysis patients and to confirm the mediating effect of treatment adherence between social support and self-care behavior. According to the results, the average of self-care behavior and treatment adherence were 3.52 and 4.01, respectively. Self-care behavior differed according to education, health status, and duration of dialysis, and treatment adherence was different in education, frequency of self-care behavior education, and social support. Self-care behavior was positively correlated with treatment adherence and social support, and treatment adherence was positively correlated with social support. Factors affecting self-care behavior were treatment adherence, social support, and health status. Factors influencing treatment adherence were self-care behavior and frequency of self-care behavior education. Further, the partial mediating effect of treatment adherence was confirmed in the relationship between social support and self-care behavior.

The self-care behavior score was higher than the 3.46 and 3.51 reported by Cho and Choe and Choi et al., respectively [4,5]. However, it was lower than the 3.61, 3.82, and 4.00 reported by Kim and Park, Song, and Choi and Choi, respectively [3,7,24]. Considering that only 17% of the participants stated that they had at least two self-care behavior education sessions per year, more self-care behavior education should be provided for outpatients of the artificial kidney unit. Regarding the sub-domains of self-care behavior, the scores were high for medication (4.13) and fistula management (4.06) and low for social adjustment (2.50) and management of blood pressure and body weight (2.94). Similar results were reported by previous studies [4,5,6,7,8]. In other words, patients easily display self-care behavior that directly impact their treatment, such as medication and fistula management. However, there is poor display of behavior that have relatively less direct impact, such as social adjustment and management of blood pressure and body weight. The low engagement in social adjustment may be attributable to difficulties in maintaining interpersonal relationships with pre-HD coworkers and friends due to the need to visit the hospital two to three times a week for HD [7]. Management blood pressure and body weight are taken frequently during hospital visits, during HD, and before and after HD; therefore, it is possible that the patients did not perceive a need to take daily measurements at home.

The mean treatment adherence score was 4.01 out of 5, which was higher than the previously reported scores such as 3.65 and 1.65 [7,8]. The high treatment adherence—a passive self-care behavior—among the participants in this study may be attributable to the fact that our participants comprised members of HD-related communities on social media who would naturally be more interested in their condition. Regarding the sub-domains of treatment adherence, the score for hemodialysis was the highest (4.75), followed by medications (4.48), diet (3.58), and fluid restrictions (3.18). Many previous studies reported that patients showed greatest adherence to hemodialysis, such as adherence to the HD schedule [8,12,13,14,15,25]. Medication adherence was the highest in the study by Natashia et al. [26]. However, Indino et al. found adherence to HD treatment to be the highest, followed by drug therapy, fluid restriction, and diet restriction [12]. These results show that despite HD having long been used as a treatment modality, patients tend to be more heavily reliant on the treatments and medications given by health care providers during their hospital visits as opposed to complying with the treatment instructions themselves. Further, patients find adhering to diet restrictions to be challenging. This is because it requires them to change their eating patterns and habits that would have been established through many years of practice [27]. Therefore, practical nursing interventions that minimize changes to the current dietary habits should be developed. These should utilize different cooking methods for patients to maintain their diet as opposed to strictly restricting the consumption of certain types of foods.

Self-care behavior significantly differed according to education, health status, and HD duration. Participants with college or higher showed higher education showed higher self-care behavior than those with middle school or lower education. The association between self-care behavior and education was consistent with previous findings [6]. Further, the association with health status was consistent with the results reported by Cho and Choe and Song [3,4]. The results were also consistent with those of Kim and Kim, who reported high medication adherence among patients with good health status [28]. Cha reported that self-care behavior was higher among patients who had been on HD for less than five years than among those who had been on HD for 5–10 years. Moreover, Choi and Choi reported that self-care behavior was higher among patients who had been on HD for less than one year, with self-care behavior decreasing with increasing HD duration [8,24]. These results are inconsistent with the present result, where self-care behavior was higher in the 1–2.99 years group than in the <1 year group. In terms of other participant characteristics, self-care behavior significantly differed according to age [6,8,26,29,30], sex [6,31,32], marital status [6,8], economic status [5], HD-related education and social support [7], primary cause of ESRD, and occupation [30]. However, some studies [3,4,33,34] observed no significant differences in self-care behavior according to participant characteristics. In addition, regardless of the frequency, self-care behavior education and self-care behavior were almost the same. However, it was confirmed that treatment adherence was higher with training at least twice a year than with no training. When checking the sub-domains of self-care behavior in Table 2, it can be seen that medication (4.13), fistula management (4.06), and management of physical problems (3.78), which are the areas corresponding to treatment adherence, had the same high scores. Other than diet (3.18), exercise and rest (3.37), and social adjustment (2.50), scores were on the low side. Although it is essential for treatment, it is an area related to lifestyle, and it is difficult to show change. In addition, it is not a short-term change, as it is an acute disease, but it may be difficult for patients to implementation because it has to be sustained over a lifetime due to the nature of a chronic disease.

Treatment adherence significantly differed according to education, frequency of self-care behavior education, and social support. Participants with college or higher education showed higher treatment adherence than those with high school or lower education. Moreover, those who received self-care behavior education at least twice a year showed higher treatment adherence than those who did not receive periodic education. Further, the high social support group demonstrated greater treatment adherence than the low social support group. The association with education was consistent with the results of Kim, who demonstrated that those who graduated from college or with higher education showed greater treatment adherence than elementary school graduates [25]. However, our findings contradicted those of Seo and Sim, who reported that elementary and middle school graduates showed greater treatment adherence than that of college graduates [35]. The inconsistency with the results of Seo and Sim may be due to the fact that participants aged ≥60 years accounted for 88% (≥70 years accounted for 47%) of their study population [35], while 89% of our study population was aged ≤64 years. Specifically, the relationship between treatment adherence and education is inconsistent, and treatment adherence does not necessarily increase with an increasing level of knowledge. Thus, interventions that aim to actually enhance treatment adherence are needed more than interventions that simply impart knowledge. In addition, 47 participants stated that they had never been educated about self-care behavior, and some of those who had been educated claimed that they had been given educational information only once during their initial HD visit. Therefore, it seems that patients should be notified that they are being educated about self-care behavior when they receive verbal instructions during their visits. The significant association between treatment adherence and social support was consistent with the findings of Krueger et al. [36]. Other influencing factors of treatment adherence included age [7,13,37,38], sex [14], income [15,25,26,39,40], marital status [15,39,40,41], occupation [25,41], and weight gain [26]. Similar to the results pertaining to self-care behavior, many studies investigated treatment nonadherence but failed to substantiate that participants’ general characteristics are consistent influencing factors of treatment adherence [16].

Regression analysis confirmed that treatment adherence, social support, and health status was significant influencing factors self-care behavior. To the best of our knowledge, no previous study investigated the relationship between treatment adherence and self-care behavior; therefore, subsequent studies should study this relationship based on our findings to confirm consistency of findings. Social support was the most potent factor influencing of self-care behavior in many studies [5,24,30,33]. Consistent with our findings, health status was identified as an influencing factor of self-care by Jeon and You [30]. Thus, measures to strengthen social support and psychosocial interventions that help patients rate their health positively may be considered when developing interventions to promote self-care behavior. The influencing factor of treatment adherence were self-care behavior and frequency of self-care behavior education.

Treatment adherence was found to have a partial mediating effect on the relationship between social support and self-care behavior. As a result of the analysis, the beta value of the indirect effect was 0.16, which was not a large value. However, it is important that a statistically significant value was confirmed as the first study to evaluate the mediating effect of treatment adherence between self-care behavior and social support. Precisely, the higher the degree of social support of the patient, the higher the treatment adherence, which could be interpreted as contributing to the increase in self-care behavior. As a result of previous studies, it was confirmed that social support was a factor influencing self-care behavior in HD patients [5,24,30,31,33], and the perceived social support of patients increases their treatment adherence [12,35]. Therefore, when developing a program to increase the patient’s self-care behavior, a higher effect could be expected if a strategy for increasing treatment adherence was included along with strengthening social support.

There are a few limitations in interpreting and generalizing the findings of this study. While the reliability and validity of the scales used in this study have been previously established, these scales may require further modification by applying them to more diverse research in a variety of experimental conditions. Moreover, we assessed self-care behavior and treatment adherence using self-reported questionnaires; therefore, the obtained results may not be an accurate reflection of the actual degree of behavior or adherence. Hence, the validity of using self-report questionnaires to assess these parameters should be tested. In addition, although the sample size was determined through power analysis, a larger sample may be needed to evaluate self-care behavior and treatment adherence more appropriately. Thus, the small sample size may limit the generalizability of our findings. Moreover, in this study, it was possible to identify factors affecting self-care behavior and treatment adherence; however, there is a limit in identifying cause-effect relationships among variables using cross-sectional study design. It is necessary to identify the cause-effect relationship among variables through future clinical trials.

## 5. Conclusions

The purpose of this study was to identify influencing factors of self-care behavior and treatment adherence in hemodialysis patients and to identify the mediating effect of treatment adherence between social support and self-care behavior. The results showed that self-care behavior of hemodialysis patients was positively correlated with treatment adherence and social support, and treatment adherence was positively correlated with social support. Factors influencing self-care behavior were treatment adherence, social support, and health status. Factors influencing treatment adherence were self-care behavior and frequency of self-care behavior education, and the explanatory power of the self-care behavior model of these three variables was 54.5%. In addition, a partial mediating effect of treatment adherence was confirmed in the relationship between social support and self-care behavior. Therefore, it is suggested that a high effect can be expected from a program that considers both treatment adherence and social support reinforcement, and this should be considered when constructing a self-care behavior intervention program in the future.

## Figures and Tables

**Table 1:** Descriptive statistics of patient characteristics *(n* = 100).

Characteristics	Categories	*n*	%	Mean ± SD
Age (years)	≤49	48	48.0	51.70 ± 9.40
	50–64	41	41.0	
	≥65	11	11.0	
Sex	Male	61	61.0	
	Female	39	39.0	
Marital status	Single	23	23.0	
	Married	77	77.0	
Education	≤Middle school	6	6.0	
	High school	43	43.0	
	≥College	51	51.0	
Occupation	Employed	45	45.0	
	Unemployed	55	55.0	
Economic status	Low	47	47.0	
	Average	33	33.0	
	High	20	20.0	
Health status	Good	29	29.0	2.92 ± 0.96
	Moderate	47	47.0	
	Poor	24	24.0	
Primary cause of ESRD	Diabetes mellitus	27	27.0	
	Hypertension	19	19.0	
	Glomerulonephritis	39	39.0	
	Others	15	15.0	
Duration of hemodialysis (years)	<1	9	9.0	7.57 ± 7.21
	1–2.99	19	19.0	
	3–9.99	47	47.0	
	≥10	25	25.0	
Frequency of self-care behavior education * (years)	none	13	13.0	8.17 ± 16.27
	1	23	23.0	
	≥2	17	17.0	
Social support	High	18	18.0	3.31 ± 0.93
	Middle	70	70.0	3.06 ± 0.89
	Low	12	12.0	3.17 ± 0.65

* Missing data. SD, standard deviation; ESRD, end-stage renal disease.

**Table 2:** Descriptive statistics of measured variables (*n* = 100).

Variables	Items	Mean ± SD	Min–Max
Self-care behavior	35	3.52 ± 0.57	1.91–5.00
Medication	2	4.13 ± 0.77	2.00–5.00
Fistula management	6	4.06 ± 0.62	2.83–5.00
Management of physical problems	11	3.78 ± 0.62	2.00–5.00
Diet	6	3.18 ± 0.71	1.00–5.00
Exercise and rest	4	3.37 ± 0.79	1.50–5.00
Management of blood pressure and body weight	3	2.94 ± 0.95	1.00–5.00
Social adjustment	3	2.50 ± 0.93	1.00–5.00
Treatment adherence	11	4.01 ± 0.48	2.78–5.00
Hemodialysis	2	4.75 ± 0.47	3.00–5.00
Medication	3	4.48 ± 0.68	2.00–5.00
Fluid restrictions	3	3.18 ± 1.38	1.00–5.00
Diet	3	3.58 ± 0.71	1.67–5.00

SD, standard deviation; Min, minimum; Max, maximum.

**Table 3:** Differences in self-care behavior and treatment adherence according to patient characteristics (*n* = 100).

Characteristics	Categories	Self-Care Behavior		Treatment Adherence	
		Mean ± SD	t or F (*p*) Scheffe	Mean ± SD	t or F (*p*) Scheffe
Age (years)	≤49	122.94 ± 23.45	0.20 (0.817)	4.00 ± 0.50	0.05 (0.950)
	50–64	124.07 ± 17.75		4.03 ± 0.47	
	≥65	119.73 ± 10.39		4.00 ± 0.48	
Sex	Male	120.48 ± 20.53	−1.62 (0.108)	3.97 ± 0.53	−0.10 (0.322)
	Female	127.07 ± 18.76		4.07 ± 0.39	
Marital status	Single	118.17 ± 24.95	−1.34 (0.185)	3.92 ± 0.44	−1.09 (0.279)
	Married	124.51 ± 18.24		4.04 ± 0.49	
Education	≤Middle school ^a^	107.33 ± 13.98	5.06 (0.008) c > a	3.82 ± 0.67	9.97 (<0.001) c > a, b
	High school ^b^	118.81 ± 22.37		3.81 ± 0.44	
	≥College ^c^	128.47 ± 16.70		4.20 ± 0.41	
Occupation	Employed	122.73 ± 19.63	−0.14 (0.887)	3.96 ± 0.39	−0.98 (0.328)
	Unemployed	123.31 ± 20.52		4.06 ± 0.54	
Economic status	Low	123.04 ± 19.07	0.01 (0.995)	3.99 ± 0.47	0.10 (0.906)
	Average	122.85 ± 23.31		4.03 ± 0.54	
	High	123.40 ± 17.14		4.04 ± 0.42	
Health status	Good ^a^	135.00 ± 20.70	9.20 (<0.001) a > b, c	3.86 ± 0.59	1.46 (0.237)
	Moderate ^b^	120.06 ± 18.36		3.43 ± 0.52	
	Poor ^c^	114.46±15.98		3.27 ± 0.46	
Primary cause of ESRD	Diabetes mellitus	120.22 ± 21.24	1.71 (0.169)	4.02 ± 0.53	0.33 (0.807)
	Hypertension	120.95 ± 17.56		3.95 ± 0.52	
	Glomerulonephritis	128.44 ± 19.78		4.06 ± 0.47	
	Others	116.80 ± 19.89		3.95 ± 0.39	
Duration of hemodialysis (years)	<1 ^a^	111.78 ± 11.80	2.82 (0.043) b > a	3.92 ± 0.53	0.46 (0.712)
	1–2.99 ^b^	132.58 ± 18.92		4.07 ± 0.41	
	3–9.99 ^c^	120.57 ± 19.85		4.05 ± 0.44	
	≥10 ^d^	124.52 ± 21.22		3.94 ± 0.59	
Frequency of self-care behavior education (years)	None ^a^	124.08 ± 21.33	0.02 (0.979)	3.91 ± 0.39	4.22 (0.020) c > a
	1 ^b^	124.48 ± 18.11		3.99 ± 0.46	
	≥2 ^c^	125.65 ± 27.83		4.35 ± 0.50	
Social support	High ^a^	131.89 ± 18.24	2.19 (0.117)	4.27 ± 0.45	3.91 (0.023) a > c
	Middle ^b^	121.03 ± 20.43		3.98 ± 0.46	
	Low ^c^	121.58 ± 17.91		3.81 ± 0.52	

^a, b, c, d^ comparison groups of Scheffe test. SD, standard deviation; ESRD, end-stage renal disease.

**Table 4:** Influencing factors on self-care behavior and treatment adherence of patients (*n* = 100).

Variables	Self-Care Behavior		Treatment Adherence	
	B (SE)	t (*p*)	B (SE)	t (*p*)
(constant)	23.13 (14.60)	1.59 (0.116)	2.10 (0.28)	7.60 (<0.001)
Education (high school)	10.00 (5.93)	1.69 (0.095)	0.17 (0.19)	0.91 (0.368)
Education (≥college)	10.70 (5.97)	1.80 (0.076)	0.35 (0.19)	1.80 (0.078)
Health status	−3.77 (1.54)	−2.45 (0.016)		
Duration of hemodialysis	−0.12 (0.19)	−0.66 (0.514)		
Frequency of self-care behavior education			0.01 (0.00)	3.47 (0.001)
Self-care behavior			0.01 (0.00)	4.34 (<0.001)
Treatment adherence	19.66 (3.31)	5.94 (<0.001)		
Social support	0.62 (0.15)	4.16 (<0.001)	0.01 (0.01)	1.54 (0.130)
F (*p*)	20.74(<0.001)		17.47 (<0.001)	
Adjusted R^2^ (%)	54.5		61.3	
Tolerance	0.21–0.99		0.19–0.97	
VIF	1.01–4.88		1.03–5.30	
Durbin-Watson	1.88		2.04	

B, unstandardized coefficients; SE, standard error; VIF, variance inflation factors.

## Data Availability

Data sharing not applicable.
